# Characteristics and Functional Relevance of Apolipoprotein-A1 and Cholesterol Binding in Mammary Gland Tissues and Epithelial Cells

**DOI:** 10.1371/journal.pone.0070407

**Published:** 2013-07-31

**Authors:** Edgar Corneille Ontsouka, Xiao Huang, Bruno Stieger, Christiane Albrecht

**Affiliations:** 1 Institute of Biochemistry and Molecular Medicine, Faculty of Medicine, University of Bern, Bern, Switzerland; 2 Swiss National Center of Competence in Research, NCCR TransCure, University of Bern, Bern, Switzerland; 3 Department of Clinical Pharmacology and Toxicology, University Hospital Zurich, Zürich, Switzerland; Emory University School of Medicine, United States of America

## Abstract

Cholesterol in milk is derived from the circulating blood through a complex transport process involving the mammary alveolar epithelium. Details of the mechanisms involved in this transfer are unclear. Apolipoprotein-AI (apoA-I) is an acceptor of cellular cholesterol effluxed by the ATP-binding cassette (ABC) transporter A1 (ABCA1). We aimed to 1) determine the binding characteristics of ^125^I-apoA-I and ^3^H-cholesterol to enriched plasma membrane vesicles (EPM) isolated from lactating and non-lactating bovine mammary glands (MG), 2) optimize the components of an *in vitro* model describing cellular ^3^H-cholesterol efflux in primary bovine mammary epithelial cells (MeBo), and 3) assess the vectorial cholesterol transport in MeBo using Transwell^®^ plates. The amounts of isolated EPM and the maximal binding capacity of ^125^I-apoA-I to EPM differed depending on the MG’s physiological state, while the kinetics of ^3^H-cholesterol and ^125^I-apoA-I binding were similar. ^3^H-cholesterol incorporated maximally to EPM after 25±9 min. The time to achieve the half-maximum binding of ^125^I-apoA-I at equilibrium was 3.3±0.6 min. The dissociation constant (K_D_) of ^125^I-apoA-I ranged between 40–74 nmol/L. Cholesterol loading to EPM increased both cholesterol content and ^125^I-apoA-I binding. The ABCA1 inhibitor Probucol displaced ^125^I-apoA-I binding to EPM and reduced ^3^H-cholesterol efflux in MeBo. Time-dependent ^3^H-cholesterol uptake and efflux showed inverse patterns. The defined binding characteristics of cholesterol and apoA-I served to establish an efficient and significantly shorter cholesterol efflux protocol that had been used in MeBo. The application of this protocol in Transwell^®^ plates with the upper chamber mimicking the apical (milk-facing) and the bottom chamber corresponding to the basolateral (blood-facing) side of cells showed that the degree of ^3^H-cholesterol efflux in MeBo differed significantly between the apical and basolateral aspects. Our findings support the importance of the apoA-I/ABCA1 pathway in MG cholesterol transport and suggest its role in influencing milk composition and directing cholesterol back into the bloodstream.

## Introduction

Like other predominantly blood borne nutrients, cholesterol crosses the mammary gland (MG) alveolar epithelium to enter milk. In neonates, rapid growth and development of tissues and organs necessitates high amounts of cholesterol, which are mainly achieved in humans through breast-feeding or bottle-feeding [1,2] (for review, see 3). However, elevated milk intake from childhood onwards may influence circulating cholesterol and represent a health risk [4,5]. For nutritional purposes, the ability to regulate the content of cholesterol in milk might offer significant benefits to people in terms of development and long-term health. However, the molecular mechanisms that mediate and control cholesterol transfer into alveolar milk are still unclear.

An accumulating body of evidence from various studies using cells other than mammary epithelial cells (MEC) suggested that the ATP-binding cassette (ABC) transporter A1 (ABCA1) orchestrates cellular cholesterol export [6–8]. It is well established that ABCA1 mediates the export of cholesterol to apolipoprotein A-I (apoA-I) as part of an energy-dependent high-density lipoprotein transport system [9,10]. Furthermore, it has been demonstrated that apoA-I binds to both ABCA1 as well as to high capacity binding sites on the plasma membrane, i.e. phospholipid rich domains [11,12]. Studies performed in fibroblasts or THP (human acute monocytic leukemia cell line), where plasma membrane has been fractionated and used for immunoprecipitation, suggested the presence of ABCA1 in non-raft, i.e. in detergent soluble domains of the plasma membrane [13–15]. The apoA-I mediated cholesterol efflux is impaired in fibroblasts from patients with mutated ABCA1 [16,17], confirming the significance of ABCA1 in regulating cellular cholesterol homeostasis. It is established that intracellular cholesterol accumulation is detrimental to cells and accelerates foam cell formation, the hallmark of cardiovascular diseases [18–20]. Whether this situation holds also true for MEC that might utilize cholesterol as a precursor molecule in the synthesis of sterol-based compounds entering the milk composition is unclear.

In the MG relatively few studies were performed with regard to the biochemistry of binding function, in contrast to characterizational studies, that simply identified the presence of ABC transporters by gene expression analysis or immunohistochemistry [21–24]. ABCA1 expression was demonstrated in the epithelium of normal and neoplastic human breast tissues [22]. The expression of ABCA1, ABCG1 and ABCA7 was shown in the alveolar and ductal epithelium as well as in mammary adipocytes [23]. More generally, ABC transport proteins, in particular ABCA1, showed differential expression in MEC and stromal cells of lactating and non-lactating bovine MG tissues with a more pronounced protein expression in MEC [23]. In MEC, ABCA1 protein was identified in the cell membrane with often apical accentuation [23]. The localization of ABCA1 in the alveolar epithelium of the bovine MG strongly suggests its importance in MG cholesterol homeostasis. On the other hand, the presence of apoA-I, the key acceptor of cholesterol exported by ABCA1, has been demonstrated in bovine milk [25,26]. Therefore, an implication of the apoA-I/ABCA1 pathway as cholesterol transport mechanism relevant for milk composition is possible, but has not been reported.

To get more insights about the role of the apoA-I/ABCA1 pathway in cholesterol transport in the MG, we sought to establish and validate a cell-based assay system capable of characterizing the kinetic determinates of cholesterol transport and efflux. The current study extends our previous work [23,24] by establishing a model using *ex vivo* collected MG tissue to define binding characteristics of components of the high-density lipoprotein (i.e. apoA-I and cholesterol), and to establish criteria and validate a cell-based cholesterol efflux assay in MEC. The binding parameters of ^125^I-apoA-I and of ^3^H-cholesterol were determined during saturation and competition binding assays with enriched plasma membrane vesicles (EPM) isolated from lactating and non-lactating bovine MG tissues. Herein, we describe the development and validation of an efficient and significantly shorter cholesterol efflux protocol that can be used for functional investigations in cultured MEC. Consequently, by applying this functional assay to MEC in the Transwell^®^ system the present study demonstrates vectorial cholesterol transport in primary MEC and thereby highlights the importance of the apoA-I/ABCA1 pathway in cholesterol transport in the MG.

## Materials and Methods

### Ethics statement

Not applicable.

### Reagents and materials

Chloramine-T trihydrate, apoA-I prepared from human plasma, cholesterol, Dulbecco’s Modified Eagle Medium (DMEM) Nutrient Mixture F-12 Ham, and RPMI medium were purchased from Sigma-Aldrich (St. Gallen, Switzerland). EGTA, HEPES, probucol, uranyl acetate, and sodium pyrosulfite were obtained from Fluka (Buchs, Switzerland). The protease inhibitor cocktail (complete EDTA-free) was purchased from Roche (Basel, Switzerland). The BCA Protein Assay Reagent kit was purchased from Pierce (Rockford, IL). The Amplex Red Cholesterol Assay kit, antibiotics, and antimycotics were purchased from LubioScience (Luzern, Switzerland).^^125^^ I (specific activity ~17Ci/mg) and 1α, 2α [N]-^3^H-cholesterol (specific activity 53Ci/mmol, in ethanol) were purchased from PerkinElmer (Schwerzenbach, Switzerland). Glass fiber filters (MN GF-3) were obtained from Macherey-Nagel (Oensingen, Switzerland). Primary bovine mammary epithelial cells (MeBo) were isolated and characterized as previously described [27] by the donator Prof. Craig Baumrucker from Penn State University (Pennsylvania, USA); RAW264.7 cells (murine macrophages) were of commercial origin (ATCC number: TIB-71) but were gifted by Prof. Jürg Gertsch from the University of Bern (Switzerland).

### A: Studies with ex vivo MG tissues

#### Tissue collection

MG tissue samples were obtained from a total of six healthy dairy cows at the slaughterhouse Marmy Viandes en Gros SA (Estavayer-Le-Lac, Switzerland) from which we obtained the permission to use these animal samples for scientific purposes. These animals were part of the routine slaughter by stunning as authorized by the Swiss Law of Animal Protection (RS 455), and have not been subjected to previous animal experimentation. Three cows were in the lactating and three in the non-lactating state. Tissues were collected immediately after slaughter. To identify the presence (or absence) of milk, and to subsequently classify the MG as lactating or non-lactating, a visual inspection of the MG incision was carried out. MG tissues were collected into ice-cold 50mM Tris HCl assay buffer (pH 7.4) containing 6mM MgCl_2_ and 1mM EGTA and supplemented with a protease inhibitor cocktail.

*Plasma membrane preparation* — The procedure for isolation of EPM was as previously described [28–30], with minor modifications described in [31]. All procedures were carried out at 4°C. In brief, MG was first minced into small pieces in chilled assay buffer. Tissues were homogenized for 2 min with an Ultra-Turrax homogenizer T25 (Janke & Kunkel, Staufen, Germany). The homogenate was centrifuged at 800 × g for 10 min followed by centrifugation of the supernatant at 10,000 × g for 10 min. The resulting supernatant was centrifuged at 100,000 × g for 1h; the obtained microsomal pellet was suspended in ice-cold assay buffer by a motor-driven Glass-Teflon homogenizer to obtain a mixed (or crude) membrane suspension. The latter was mixed with MgCl_2_ (final concentration 12mM) under constant stirring for 30 min, and then centrifuged at 3000 × g for 15 min. In this study, MgCl_2_ was used instead of CaCl_2_ as described by Lin and colleagues [31], because millimolar concentrations of calcium might alter the overall structure and integrity of membranes [32,33]. Following MgCl_2_ treatment, the supernatant containing plasmalemma was centrifuged at 48,000 × g for 1h. The pellet was re-suspended in assay buffer, and the resulting suspension, i.e. EPM was stored at -80°C until used. The enrichment of plasma membrane preparations was confirmed by Western blot analysis of ABCA1, where a stronger ABCA1 reactivity in EPM as compared to the crude membrane preparation was observed (unpublished data).

#### Transmission electron microscopy

Fixation and processing were carried out as described by 34. Ultrathin (~ 80 nm) sections of embedded samples were cut with a ultramicrotome UC6 Leica Microsystems (Vienna, Austria) and contrasted with lead citrate and uranyl acetate. The stained sections were inspected with a transmission electron microscope CM12 Philips (Eindhoven, Netherland) equipped with a digital camera Morada, Soft Imaging System (Münster, Germany) and image analysis software (iTEM) at various magnifications.

#### Biochemical analyses

The protein concentration of EPM suspensions was determined with a BCA kit. The cholesterol content of the EPM and the cell lysate as well as the cholesteryl ester content of the cell lysate (see section B below) were measured with Amplex Red^®^ Cholesterol Assay kit. All analyses were performed following the manufacturers’ protocols.

#### Radiolabeling of substrates

ApoA-I was iodinated with ^125^I by using the chloramine-T method [35]. In brief, apo-AI was diluted in phosphate buffer and then mixed with 0.5mCi of ^125^I. The iodination reaction was initiated by adding chloramine-T trihydrate to the mixture, and was stopped 30 sec later with sodium pyrosulfit. The reaction mixture was filtrated with Sephadex G-200 superfine Pharmacia Fine Chemicals (Upssala, Sweden) poured onto a 1.6×33cm column for desalting and removal of free ^125^I in a buffer consisting of 10 mM Tris-HCl, 100 mM KCl, 1mM sodium azide, pH 7.4 that was supplemented with 2mg/ml of bovine serum albumin (BSA) to prevent the loss of the protein due to unspecific binding to the column [36]. The specific activity of ^125^I-apoA-I was 41µCi/µg protein.

#### Binding studies and procedures

Binding assays were performed with working solutions of ^3^H-cholesterol and ^125^I-apoA-I that were prepared by diluting their respective stock solutions in Tris-HCl assay buffer. If not otherwise indicated, all binding assays were performed with a fixed amount (100µg) of EPM protein at 37°C. The final concentration of ethanol in the binding assay mixture was < 0.1%.

The association binding (or incorporation) of ^3^H-cholesterol (1nM and 10nM) and of ^125^I-apoA-1 (10nM) to EPM was determined by incubating the assay mixture for different durations up to 48h. To study the dissociation binding of ^125^I-apoA-I, the radiolabel (10nM) was first incubated with EPM until the equilibrium was reached; then 1.4µM of unlabeled apoA-I was added to the mixture followed by different incubation times. The saturation binding of ^125^I-apoA-I was analyzed by measuring the binding of increasing concentrations of radiolabel (range 0.5 to 55 nM) to EPM for 15 min in the presence and absence of 1.4µM unlabeled apoA-I. To verify that ^125^I-apoA-I binding (10nM) can be inhibited, its binding to EPM for 15 min in the presence and absence of 1.4µM unlabeled apoA-I was measured and compared. In addition, the inhibition binding of ^125^I-apoA-I by increasing concentrations (10^-13^ to 10^-4^M) of the ABCA1 inhibitor probucol [37,38], used as a complex with BSA [38], was determined. Furthermore, the likely interference of cholesterol on apoA-I binding was analyzed by measuring the binding of 10nM ^125^I-apoA-I to EPM for 15 min in the presence and absence of preloading with 1.6mM cholesterol for 30 min at 37°.

All binding assay mixtures were incubated under constant shaking, and reactions were stopped by adding 2ml of chilled assay buffer (the same as for tissue collection). The mixtures were then filtrated through glass fiber filters MN GF-3 (Macherey-Nagel, Oensingen, Switzerland) by using a vacuum filtration manifold (Hölzel, Wörth, Germany). Prior to use, the filters were equilibrated in Tris-HCl assay buffer supplemented with 2 mg/ml (w/v) BSA.^^125^^ I-activity and ^3^H-activity were measured with a γ-counter and β-counter, respectively (Kontron, Schlieren, Switzerland). The GraphPad software program (GraphPad Software, Inc., San Diego, CA) was used for curve fitting and for the determination of binding characteristics of ^125^I-apoA-I and ^3^H-cholesterol.

### B: Cell culture studies

#### Cell culture

MeBo cells originating from two dairy cows at late lactation have been previously characterized [27,39]. Cells were incubated at 37°C with 5% CO_2_ in T75 polystyrene culture flasks. They were grown in complete DMEM-F12 medium supplemented with 10% fetal bovine serum and 1% antibiotics/antimycotics. For cell splitting and passaging, 0.05% trypsin EDTA solution was used. To assure a similar differentiation state, all efflux experiments were performed with MeBo cells within two passage numbers originating from the same batch. Throughout the experiments the cell density was approximately of 200’000 cells per well in 12-well plates. The confluence prior to the start of the cholesterol efflux assay was approximately 90%.

#### Cholesterol efflux

The cholesterol efflux assay was adapted from a previously published procedure for RAW264.7 cells [40]. Prior to using the assay in MeBo cells the protocol was tested in RAW264.7 cells cultured in complete RPMI medium. Based on the binding characteristics of cholesterol and apoA-I obtained from the ex vivo investigations (see Results, section A), MeBo cells growing in complete DMEM-F12 medium on the plastic surface were loaded for 0.5, 1 and 24h with ^3^H-cholesterol (1µCi/ml, dissolved in ethanol). ^3^H-cholesterol uptake by cells was estimated by relating the remaining ^3^H-activity in the medium (M1) to the initially loaded radioactivity (uptake evaluation 1). After cholesterol loading cells were equilibrated for 0, 0.5, 1 and 18h in serum-free DMEM-F12 medium. Cholesterol efflux was initiated by adding the cholesterol acceptor apoA-I to the cell medium; the efflux medium (M2) was collected after apoA-I incubation for 0.25, 1 and 4h. After removal of the efflux medium the plates were frozen at -20 °C for 30 min. Then, dPBS was added and the plates were shaken for 30 min at room temperature prior to lysate collection. The collected M1 and M2 samples were centrifuged for 10 min to get rid of cell debris. An equal volume of M1, M2, and cell lysate was transferred into scintillation vials and mixed with 4ml of the scintillation liquid for β-counting.

The percentage of ^3^H-cholesterol efflux was calculated by relating the radiolabel in M2 to the sum of radiolabel in M2 and in cell lysate. ApoA-I mediated cholesterol efflux was obtained by subtracting the value of the efflux measured in the absence of apoA-I from that in the presence of apoA-I. The cholesterol uptake was furthermore evaluated by calculating the sum of the radiolabel in the cell lysate and in M2, and related to the initially loaded radioactivity (uptake evaluation 2).

#### *Vectorial* *cholesterol* efflux using the Transwell^®^ system

To distinguish apical from basal cholesterol transport, MeBo cells were cultured in double chamber Transwell^®^ plates. Cells were grown to confluence on six-well cell culture Transwell^®^ plates (BD Biosciences, La Pont de Claix, France) in DMEM-F12 medium supplemented with 10% FBS and 1% antibiotics/antimycotics added to the top (apical) and bottom (basal) chambers. Cells were grown for approx. five days until reaching confluence. The formation of a tightly sealed polarized cell monolayer at confluence was verified by measuring the resistance and subsequently calculating the trans-epithelial electrical resistance (TEER) in cell-loaded and cell-free Transwell^®^ membranes with the Millicell-ERS Volt-Ohm meter (Millipore, MA, USA) according to [41]. Lucifer Yellow dilithium salt (Sigma, Switzerland), a fluorescent dye mainly transported across polarized cells in a paracellular fashion, was used to monitor the tight junction integrity [42]. The apparent permeability (P_app_) through the cell-loaded and cell-free Transwell^®^ membranes was calculated as described by others [43]. Fluorescence detector Flex Station II plate reader (Molecular Devices GmbH, Biberach, Germany) was used to measure fluorescence at an excitation and emission wavelength of 425nm and 530nm, respectively. The appearance of fluorescent Lucifer Yellow is proportional to the amount of the dye crossing the MEC monolayer. After loading to the apical and basal compartment, respectively, Lucifer Yellow was measured in the opposite chamber. The procedure for the efflux was as described above (loading 1h, equilibration 1h, efflux 1h), except that apoA-I was loaded to the apical and the basal chambers.

### Statistical analysis

All statistical analyses were performed with non-parametric tests using GraphPad Prism (San Diego, CA) software. Protein and cholesterol content of EPM, maximal binding capacity of ^125^I-apoA-I, determinants of apoA-I mediated efflux and vectorial apoA-I mediated ^3^H-cholesterol efflux in MeBo cells were analyzed for statistical difference using the Mann-Whitney test; cholesterol uptake and efflux at various time points were compared using the Kruskal-Wallis test. The level of significance was set at *P* < 0.05.

## Results

The unequivocal reproduction of lactating and non-lactating states of the MG in vitro is difficult due to the complexity in the regulation of pregnancy-lactation cycle as well as to factors inherent to the cell culture. Therefore, a two-step analytical approach combining *ex vivo* MG tissues and culturing of MEC had been chosen to ascertain both the suitability of the defined cholesterol efflux conditions for functional studies with primary MEC and the relevance of the apoA-I/ABCA1 pathways in cholesterol transport in the MG.

### A: Studies using ex vivo MG tissues

**Figure 1:**
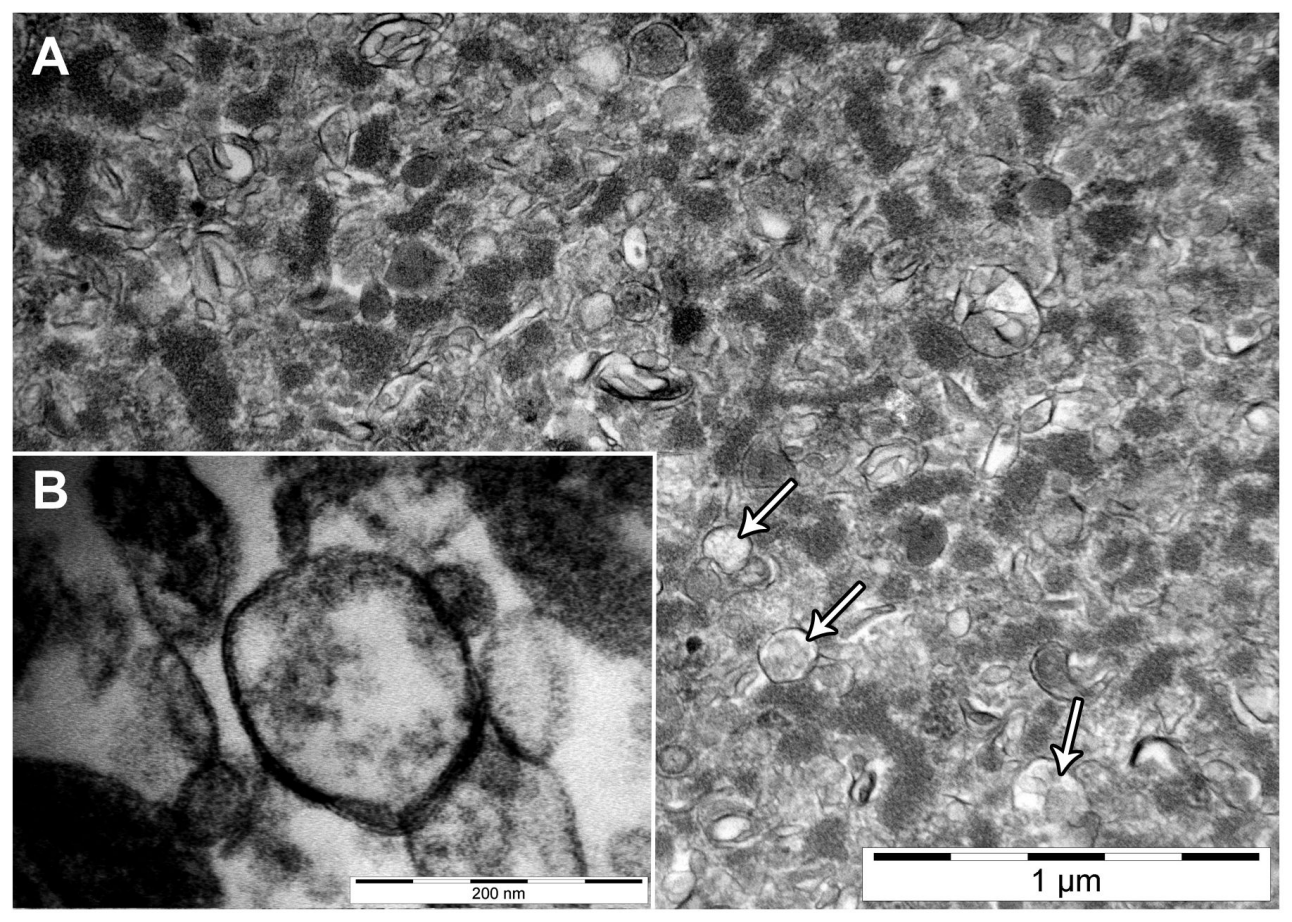
Transmission electron microscopy of mammary gland (MG) enriched plasma membrane vesicles (EPM). A: Representative electron micrograph of EPM from lactating MG at 31’000 × magnification. Arrows depict single vesicles. B: The bilayer structure of the EPM from the same lactating MG at 230’000 × magnification. Electron micrographs of EPM isolated from non-lactating MG (not shown) were similar to that of lactating tissue.

^*3*^ *H-cholesterol incorporation to EPM* — The incorporation of ^3^H-cholesterol to EPM reached a plateau after 25 ± 9 min both when 1nM or 10nM ^3^H-cholesterol was used (Figure 2). The average percentage of ^3^H-cholesterol incorporation was markedly decreased at the 10 fold higher concentration of ^3^H-cholesterol (Figure 2), with average values of 92 ± 6% (1nM) as compared to 61 ± 8% (10nM).

*Isolation and identification of EPM* — The amounts of isolated EPM differed depending on the physiological stage of the MG (Table 1). The total protein levels in EPM were higher in lactating than in non-lactating MG tissues. In contrast, cholesterol content of EPM was higher in non-lactating MG than in lactating MG tissues (Table 1). The isolated EPM vesicles were inspected by electron microscopy (Figure 1). Figure 1A shows a representative image of EPM vesicles derived from lactating MG tissues (31’000 x magnification). The insert (Figure 1B) depicts a bilayer structure of the same EPM sample. Similar images were obtained for EPM isolated from non-lactating MG (not shown).

**Table 1:** Biochemical characteristics of mammary gland derived enriched plasma membranes (EPM).

**Traits**	**Lactating tissues**	**Non-lactating tissues**
**EPM proteins^1^**		
*mg per g MG tissue*	1.32 ± 0.28^a^	0.77 ± 0.27^b^
**Cholesterol content^2^**		
*µmol per mg EPM protein*	0.20 ± 0.01	0.31 ± 0.12
*µmol per g MG tissue*	0.26 ± 0.02	0.23 ± 0.09
**^125^I-apoA-I binding^3^**		
*B*_*max*_ * (pmol/mg protein)*	5.87 ± 1.88^b^	11.5 ± 1.19^a^
*K*_*D*_ * (nmol/L)*	40 ± 24	74 ± 12
*% inhibition by cold apoA-I*	71.4 ± 8.29	79.2 ± 2.59
*% increase by cholesterol*	64 ± 24	40 ± 23

All results are based on enriched plasma membranes (EPM) prepared and processed as described in *Materials and Methods*. Data are presented as mean ± SD (n=3).

1 Protein concentrations of EPM were measured with the BCA protein assay kit

2 Cholesterol content of EPM was determined by using Amplex Red Cholesterol Assay kit following the manufacturer’s instructions.

3 All binding reactions were incubated for 15 min at 37°C under constant shaking.

The maximal binding capacity (B_max_) and the dissociation constant (K_D_) of ^125^I-apoA-I binding were measured during saturation binding of ^125^I-apoA-I (range 2 to 56nM) to 100µg EPM as presented in Figure 3B. The specific binding of ^125^I-apoA-I was obtained by subtracting binding in the presence of cold apoA-I (1.4µM) from that in the absence of cold apoA-I. The percentage inhibition of ^125^I-apoA-I binding (10nM) by cold apoA-I was obtained by relating the binding of ^125^I-apoA-I in the presence of cold apoA-I to the binding in the absence of cold apo-A1, which was defined as 100%.

The effect of cholesterol loading on ^125^I-apoA-I binding was determined by comparing ^125^I-apoA-I binding to EPM (100µg) in the presence and absence of preloading with 1.6mM cholesterol for 30 min at 37°C.

Mean values with different superscript letters (a b within the row are statistically different (*P* < 0.05).

**Figure 2:**
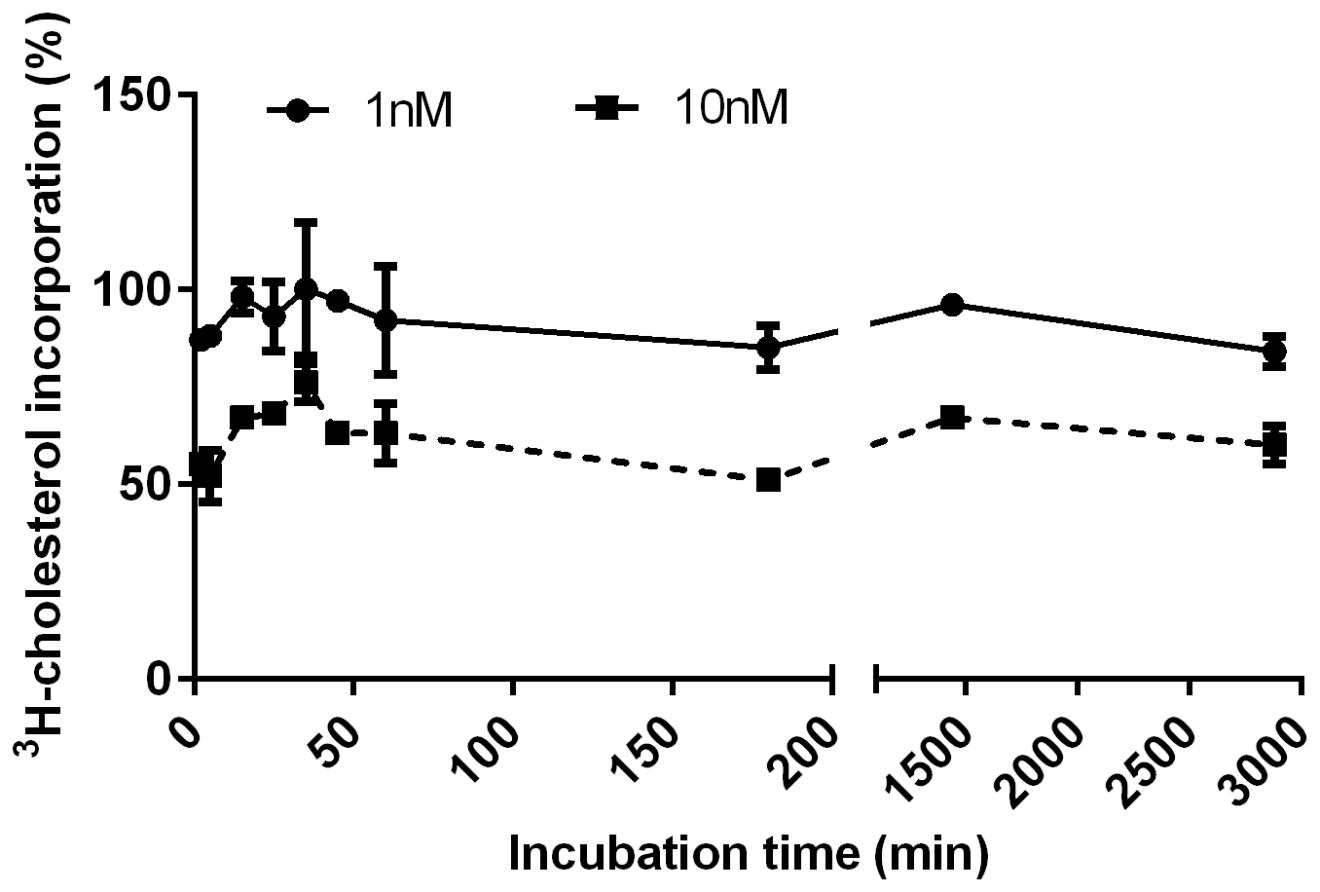
Time-dependent ^3^H-cholesterol incorporation to mammary gland (MG) enriched plasma membrane vesicles (EPM). The figure illustrates representative kinetics of incorporation of 1nM (●) and 10nM (■) ^3^H-cholesterol into EPM (100µg) isolated from lactating MG tissues. Data represent the means of three independent experiments performed in triplicates. The incorporation reaction was incubated at 37°C using glass tubes coated with bovine serum albumin. The radioactivity of the filter was measured using a β-counter. No difference was found between lactating and non-lactating MG.

**Figure 3:**
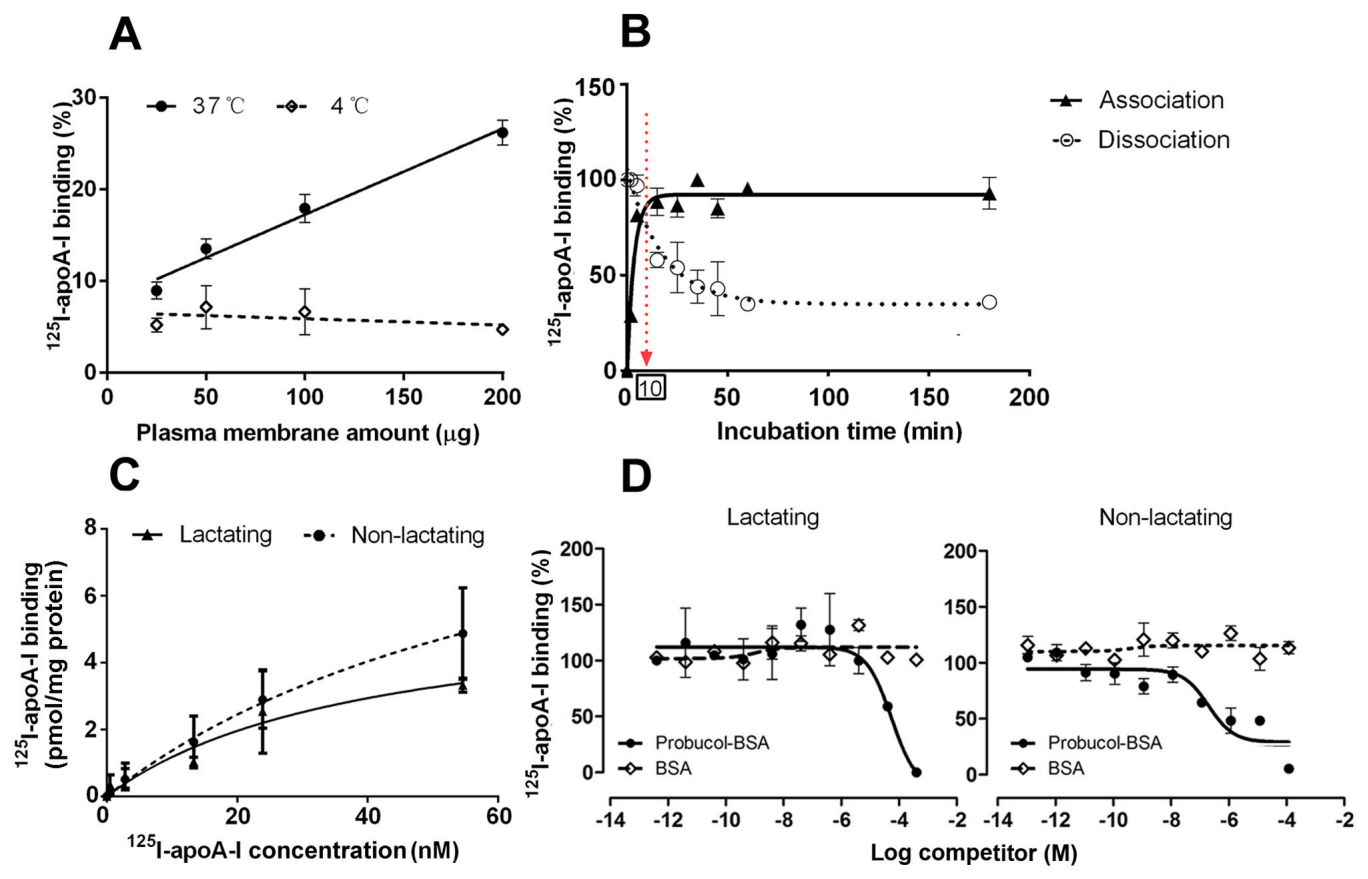
Binding of ^125^I-apoA-I to mammary gland (MG) enriched plasma membrane vesicles (EPM). A: Representative graph of ^125^I-apoA-I binding (5nM) to increasing concentrations of EPM (range 0.25 to 2 mg/ml) at 37°C (●) and 4°C (□). Dose-dependent ^125^I-apoA-I binding was only observed at 37°C. B: Representative curves of ^125^I-apoA-I binding (10nM) kinetics at 37°C to a fixed amount (100µg) of EPM. For the association binding of ^125^I-apo-A1 (▲), the maximal binding (saturation) was reached after 10 min incubation at 37°C, and was expressed as 100% binding. For the dissociation binding (○), ^125^I-apoA-I binding was incubated for 15 min at 37°C. Then, excess amounts (40µg/ml) of cold apoA-I were added and the dissociation of ^125^I-apoA-I was evaluated at indicated incubation times. Data shown are from lactating MG. Similar curves were obtained for non-lactating MG. C: Saturation binding curve of ^125^I-apoA-I (range 2 to 56nM) to a fixed amount (100µg) of EPM from lactating (▲) and non-lactating (●) MG tissues. The reaction was incubated for 15 min at 37°C. D: Competition binding of ^125^I-apoA-I to a fixed amount of EPM (100µg) from lactating and non-lactating MG tissues by probucol-BSA (●) and BSA (◊). The probucol-BSA complex was prepared as described by others (37). The reaction was incubated for 15 min at 37°C. All other details of the binding procedure were as described in Figure 2 except that the radioactivity of the filters was measured with a γ-counter. All data are expressed as means ± SD.

*Linearity of* ^^125^^ *I-apoA-I binding* — The binding of ^125^I-apoA-I at 37°C increased with augmenting amounts of EPM (R^2^ = 0.98) independent of the physiological state of the MG (Figure 3A). In contrast, no increase of ^125^I-apoA-I binding (R^2^ = 0.22) was observed when the reaction was incubated at 4°C (Figure 3A). Results illustrated in Figure 3A are representative data derived from non-lactating MG tissues. Similar data were obtained for lactating MG (not shown).

#### Association and dissociation binding of ^^125^^I-apoA-I

The binding of ^125^I-apoA-I reached maximal values after 10 min incubation at 37°C (Figure 3B), and did not change with a prolonged incubation period. Half maximal ^125^I-apoA-I association binding to EPM was 3.3 ± 0.6 min (Figure 3B) regardless of the physiological state of the MG tissue.^^125^^ I-apoA-I dissociation binding was 25 ± 3 min (Figure 3B). A fraction of ^125^I-apoA-I binding ranging between 30–36% could not be inhibited by excess amounts of cold apoA-I (Figure 3B). The association and dissociation of ^125^I-apoA-I did not change with an incubation period until 48h (data not shown).

#### Saturation binding of ^^125^^I-apoA-I

Although the binding of ^125^I-apoA-I did not clearly saturate within the range of concentrations used, apparent K_D_ and maximal binding capacity (B_max_) can be calculated from the fitting curve, assuming saturation at higher doses. The average B_max_ values of ^125^I-apoA-I binding derived from the fitting curve of the three experiments differed between non-lactating (95% confidence interval: 1.6, 21) and lactating MG tissues (95% confidence interval: 2, 10) (Figure 3C and Table 1). In both cases, K_D_ values derived from ^125^I-apoA-I saturation binding curves were in the nanomolar range (Table 1).

#### Competition binding of ^^125^^I-apoA-I

The binding of ^125^I-apoA-I was inhibited by excessive amounts of unlabeled apoA-I in EPM from both lactating and non-lactating MG tissues (Table 1). Furthermore, increasing concentrations of probucol-BSA (10^-13^ to 10^-4^M) inhibited ^125^I-apoA-I binding at micromolar concentrations in lactating and non-lactating MG tissues (Figure 3D). Binding data were fitted to a one-site inhibition model (Figure 3D) and EC_50_ values derived from the fitting curve of lactating (R^2^ = 0.77) and non-lactating (R^2^ = 0.81) EPM were 13 ± 10 and 0.4 ± 0.03 µM, respectively. BSA alone did not inhibit ^125^I-apoA-I binding (Figure 3D).

#### Interference of cholesterol with ^^125^^I-apoA-I binding

Loading of EPM with 1.6mM cholesterol (dissolved in 100% ethanol) markedly increased ^125^I-apoA1 binding in lactating and non-lactating MG tissues (Table 1). Additional investigations showed that cholesterol loading markedly increased the EPM cholesterol content (unpublished data).

### B: Cell culture studies

Taking into account the binding characteristics of ^125^I-apoA-I and ^3^H-cholesterol to EPM obtained in the ex-vivo investigations (see Results, section A), we optimized the cellular cholesterol efflux in MeBo cells with regard to incubation, equilibration and efflux times. The initial protocol for cellular cholesterol efflux was based on RAW264.7 cells (murine macrophages) and yielded efflux values of 10.6 ± 2.27% for 4h, i.e. similar to the results reported by the authors [40].

#### Uptake profile of ^3^H-cholesterol by MeBo

**Figure 4:**
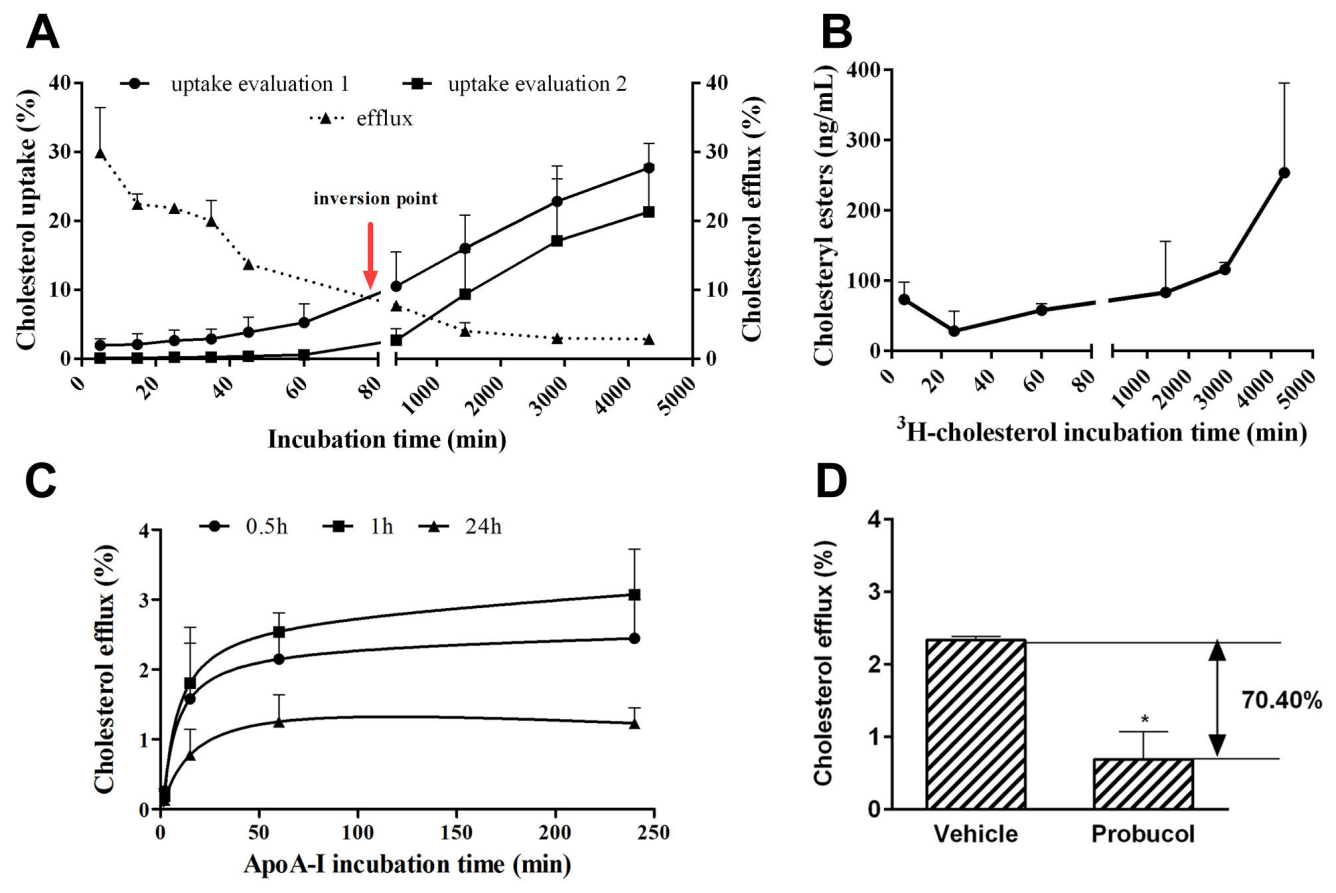
Kinetics of ^3^H-cholesterol transport in primary bovine mammary epithelial (MeBo) cells. **A**: Comparative uptake (●) and efflux (▲) of ^3^H-cholesterol by MeBo cells growing as a monolayer in DMEM-F12 medium supplemented with 10% fetal bovine serum and 1% antibiotics/antimycotics. Cholesterol efflux was performed in the presence of 10µg/ml apoA-I (details see Materials and Methods). The cholesterol uptake was calculated either based on the amount of radiolabel disappearing from the medium (evaluation 1) or on the sum of the radiolabel measured in the cell lysate and efflux medium (evaluation 2). Both values were related to the initially loaded amounts of radiolabel that was defined as 100%. The arrow depicts the inversion point that is the incubation time where cholesterol uptake and efflux are in apparent equilibrium. It represents a threshold beyond which the availability of ^3^H-cholesterol for efflux becomes markedly reduced in favor of increasing intracellular compartmentalization.” **B**: Cholesteryl ester content of the cell lysate. Cholesteryl esters were measured with the Amplex Red^®^ assay kit according to the manufacturer’s instructions. All experimental details were as described in section A. **C**: Time-dependent saturation curve of apoA-I mediated efflux. Cells were loaded with cholesterol for 0.5h (●), 1h (■), and 24h (▲). Details for cell equilibration were as described in section A. Please note that in contrast to Figure 4A, the background efflux measured in the absence of apoA-I was recorded, and subtracted from the total efflux measured in the presence of 10µg/ml of apoA-I. **D**: Regulation of apoA-I mediated efflux in MeBo cells. Cells were loaded with ^3^H-cholesterol (1µCi/ml) in complete DMEM-F12 medium supplemented with 10% fetal bovine serum and 1% antibiotics for 24h. Cells were equilibrated for 18h in serum-free medium followed by the efflux in the presence of apoA-I (10µg/ml) for 4h (see Materials and Methods for additional details). Cells were treated with probucol, an inhibitor of ABCA1, throughout the efflux time. All data are expressed as means ± SD of three independent experiments performed in triplicates.

As described in *Materials and Methods* cholesterol uptake was calculated in two different ways: firstly estimated from the amount of radiolabel disappearing from the medium (M1, uptake evaluation 1) and secondly calculated as the sum of the radiolabel measured in the cell lysate and efflux medium M2 (uptake evaluation 2). Both calculation methods showed a steady increase of ^3^H-cholesterol uptake with incubation time (Figure 4A). The inversion point shows the cholesterol incubation time where uptake and efflux are apparently in the equilibrium (Figure 4A). In parallel, intracellular cholesteryl esters accumulated with increasing incubation times (Figure 4B).

The percentage of uptake was lower when cells were loaded with ^3^H-cholesterol for 0.5h than for 24h (Table 2). However, the percentage of uptake did not change between cells loaded for 0.5h and 1h, and for 1h and 24h, respectively (Table 2).

**Table 2:** Comparative ^3^H-cholesterol uptake and efflux in primary bovine mammary epithelial cells.

	**Incubation time for cholesterol or apoA-I**					
**Traits**	2 min	15 min	30 min	1h	4h	24h
**Uptake** ^1^ (%)	n.d.	n.d.	17 ± 8^b^	21 ± 8^ab^	n.d.	30 ± 9^a^
**Efflux** ^2^ (%)	0.17 ± 0.10^c^	1.32 ± 0.64^b^	n.d.	1.91 ± 0.65^ab^	2.15 ± 0.88^a^	n.d.

Data (mean ± SD) are representative of three independent experiments performed in triplicates. Mean values with different superscript letters (a b c within the row are statistically different (*P* <0.05).

^1^ shows the uptake of ^3^H-cholesterol after loading cells with 1µCi/ml of ^3^H-choelsterol for 30 min, 1h and 24h. The uptake is indirectly calculated by measuring the remaining radioactivity after each incubation time. The initially loaded activity was defined as 100%.

^2^ shows apoA-I mediated efflux that was obtained by subtracting the background efflux (in the absence of apoA-I) from total efflux (in the presence of 10µg/ml apoA-I). Cells were loaded with ^3^H-cholesterol for 30 min, 1h and 24h in complete DMEM-F12 medium and equilibrated for 18h in serum-free DMEM-F12 medium. Pooled data of apoA-I mediated efflux are shown as no differences were observed between different ^3^H-cholesterol loading times (30 min, 1h and 24h).

n d: not determined.

^*3*^ *H-cholesterol efflux* — ApoA-I mediated ^3^H-cholesterol efflux was unchanged when the apoA-I incubation time was 1 or 4h (Table 2). An apoA-I incubation time of only 2 min significantly decreased ^3^H-cholesterol efflux as compared to all other time points. However, there were no differences in cholesterol efflux between apoA-I incubation times of 15 min and 1h (Table 2). The apoA-I mediated cholesterol efflux in MeBo cells showed always a saturable pattern (Figure 4C).

Given that the probucol-BSA complex inhibited ^125^I-apoA-I binding to ex vivo isolated EPM in µmolar concentrations, the effect of probucol treatment (10µM) on cellular cholesterol efflux was analyzed. ApoA-I mediated ^3^H-cholesterol efflux was reduced by 70.4% in probucol treated as compared to control cells (Figure 4D).

#### Vectorial ^3^H-cholesterol efflux

**Figure 5:**
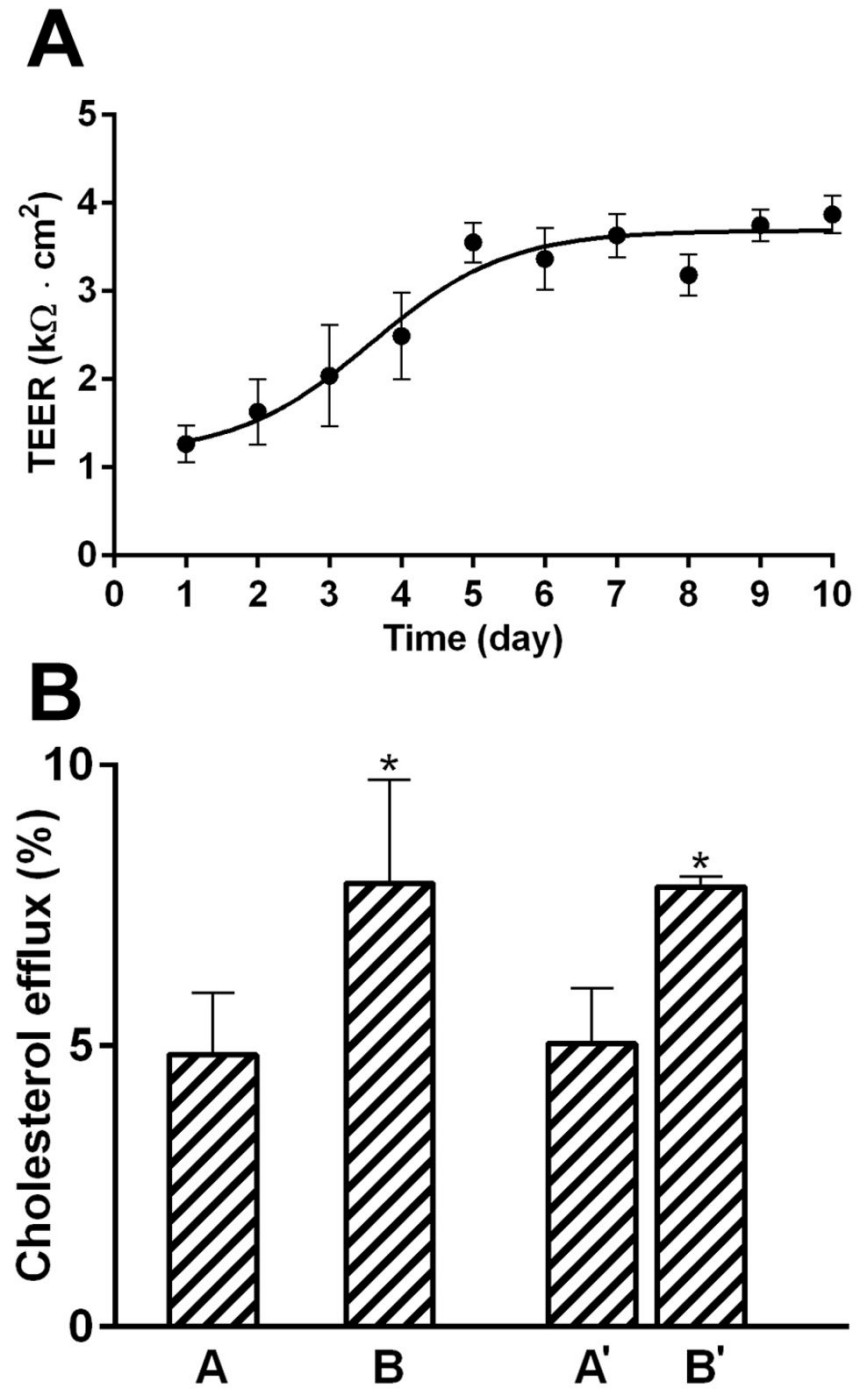
Vectorial ^3^H-cholesterol transport in primary bovine mammary epithelial (MeBo) cells. A: Time-dependence of the trans-epithelial electrical resistance of MeBo cells grown as monolayer in Transwell^®^ tissue culture plates. MeBo cells were exposed to DMEM-F12 medium supplemented with 10% fetal calf serum and 1% antibiotics/antimycotics that was added to the apical and basal chambers. Resistance was measured according to the manufacturer’s instructions in quadruplicates of >12 wells. Trans-epithelial electrical resistance was calculated according to [41]. B: Vectorial apoA-I mediated ^3^H-cholesterol efflux in MeBo cells. The experiment was performed according to the optimized protocol (loading 1h, equilibration 1h, efflux 1h). All other details of the procedure were as described in Figure 4B. ApoA-I was added either to the apical (A) or to the basal (B), or to both chambers (A’, B’). ApoA-I mediated cholesterol efflux was calculated separately for the apical and the basal chamber by subtracting the background efflux. All data are expressed as means ± SD of triplicates measurements.

The evaluation of TEER indicated that MeBo cells formed a tightly sealed monolayer after approx. 5-7 days of culture in complete medium (Figure 5A). In addition, the permeability test with Lucifer Yellow confirmed the presence of a tightly sealed monolayer (P_app_< 10^-6^ cm/s). Using the optimized efflux protocol (loading 1h, equilibration 1 h, efflux 1h), apoA-I mediated cholesterol efflux occurred at both the apical and basal side of the MeBo monolayer, but was more pronounced at the basal side (Figure 5B). Simultaneous loading of apoA-I to both chambers gave similar results as individual loading to the apical and basolateral compartment, respectively (Figure 5B, A’ and B’). The significantly higher cholesterol efflux at the basolateral side was also confirmed when the conventional efflux protocol (loading 24h, equilibration 18 h, efflux 4h) was applied (data not shown).

## Discussion

The present study shows that EPM isolated from *ex vivo* MG tissues are suitable for defining the binding characteristics of apoA-I and cholesterol and that those criteria are useful for designing optimal cholesterol efflux assay conditions applicable to primary MEC. The binding characteristics of apoA-I and cholesterol were tested in EPM extracted from MG tissues (i.e. EPM originating from various cell types) at native lactating and non-lactating states to verify that the finally defined efflux conditions can be translated to pure MEC independent of their naturally or experimentally induced physiological state (lactating or non-lactating). In this context it is worthwhile to note that the physiological interpretation of the comparison between lactating and non-lactating MG was beyond the scope of the present study.

The identification of ^125^I-apoA-I binding to EPM isolated from lactating and non-lactating tissues supports the importance of apoA-I mediated cholesterol transport in the MG. The binding of ^125^I-apoA-I to EPM was fast and temperature sensitive because it reached the plateau after 10 min incubation, and occurred in a concentration dependent manner at 37°C but not at 4°C. These findings are in agreement with previously reported apoA-I binding data [44,45]. In the current study, the time to achieve the half-maximum binding of ^125^I-apoA-I at equilibrium was 3.3±0.6 min, whereas the half-time of dissociation binding was 25 min. The data reported here corroborate those published by others in 293 cells using cross-linking assays [46], suggesting that the binding properties of iodinated apoA-I were similar between the two studies. The fact that ^125^I-apoA-I binding reached a plateau after only approximately ten minutes suggests that an apoA-I incubation time of a few minutes, instead of several hours as frequently used in efflux experiments [7], is sufficient for cholesterol efflux in primary MEC.

Interestingly, the binding of ^125^I-apoA-I to EPM was displaced at µmolar concentrations by probucol, an inhibitor of ABCA1 [37,38,47]. Accordingly, the apoA-I mediated cholesterol efflux by MeBo cells was strongly suppressed in cells treated with probucol used at comparable concentrations. Taken together, these findings support a role of the apoA-1/ABCA1 pathway in cholesterol transport in the MG.

In the current study the binding of ^125^I-apoA1 to EPM was increased when the latter was loaded with millimolar concentrations of cholesterol. On the other hand, cholesterol loading increased the EPM cholesterol content. Taken together this may suggest a potential role of cholesterol as a “modulator” of apoA-I binding. It may be speculated that loaded cholesterol contributes to the formation of additional lipid-rich domains to which apoA-I binds. In support of this assumption, we found that the maximal binding of ^125^I-apoA-I (normalized to EPM protein) tended to be greater in non-lactating MG tissues containing higher levels of cholesterol than in lactating MG tissues with lower cholesterol content. Interestingly, if the binding data are normalized to the amount of EPM cholesterol, the maximal binding capacity of ^125^I-apoA-I was similar between lactating and non-lactating MG (unpublished data). In the current study a portion of ^125^I-apoA-I binding could not be displaced by native apoA-I. Similar findings have been previously reported in other studies where it was speculated that the presence of iodine in the apoA-I molecule may cause changes in the phospholipid binding properties [45] [48]. In the present study it was not determined if iodine incorporation occurred at the region where apoA-I binds to ABCA1. Nonetheless, as discussed above, the half-time of association and dissociation of ^125^I-apoA-I binding reported here were similar to that determined by others.

In the present study the initially determined binding characteristics of ^3^H-cholesterol and ^125^I-apo-AI in the *ex vivo* MG model served to optimize the cholesterol efflux conditions in MEC. The rationale for the optimization was as follows: 1) ^3^H-cholesterol incorporation to EPM reached the plateau after less than 1h incubation at 37°C whereas ^3^H-cholesterol uptake by MeBo cells steadily increased with incubation time. These results imply that EPM *per se* have a limited cholesterol loading capacity that might be reached relatively fast. 2) The inversion point was observed in cells loaded for approximately 80 min. This point seemed to be a threshold beyond which the availability of ^3^H-cholesterol for efflux becomes markedly reduced in favor of increasing intracellular compartmentalization likely in the form of cholesteryl esters. Based on that, a preloading step lasting for 1 h could be sufficient for the cholesterol efflux assay in MEC. A similar loading time has been utilized for efflux assay in J774 cells using BODIPY-cholesterol [49]. 3) The inversion point showed an apparent equilibrium between cholesterol efflux and uptake processes. The comparison of cell equilibration times lasting 0, 0.5, 1, and 18h suggested that an equilibration time of 1h is optimal for cholesterol efflux in MeBo cells. 4) The concentration of apoA-I typically used for the efflux (10µg/mL) is more than 4 times its measured K_D,_ and is therefore high enough to favor maximal apoA-I activity.

In summary, the herein optimized cholesterol efflux protocol for MeBo cells includes loading with ^3^H-choleserol (1µCi/ml) for 1h, cell equilibration in serum-free medium for 1h, and cholesterol efflux in the presence of 10µg/mL apoA-I for 1h. This protocol allows performing the cholesterol efflux assay within a time period of 3h instead of 46 h needed with the protocol initially published by others [40]. However, the currently optimized protocol in MeBo cells did not show the same efficiency in RAW264.7 cells. We observed that both cholesterol uptake and efflux in RAW264.7 were higher when cells were loaded for 24h than for 1h. In MeBo cells cholesterol uptake was higher when cells were loaded for 24h than for 1h, but contrary to RAW264.7, the efflux levels remained similar (unpublished data). This might be due to differences related to cholesterol processing in RAW264.7 and MEC. Another limitation of the developed short cholesterol protocol might arise when specific protein modulating agents (e.g. ABCA1 inducers or inhibitors) are applied which may need longer than one hour for exerting measurable effects on protein function.

Finally, this optimized cholesterol efflux protocol allowed us to functionally study the main features of vectorial cholesterol transport in cultured MEC. When the cholesterol efflux assay was applied to MeBo cells in the Transwell^®^ system, we were able to show that the apoA-I/ABCA1 pathway mediates cholesterol efflux from both the apical (milk-facing) and basolateral (blood-facing) side. At steady state conditions, i.e. in complete culture medium and the absence of hormonal stimuli, cholesterol efflux appeared to be more accentuated at the basolateral aspects of MeBo cells. Further studies have to clarify whether pregnancy-related and/or lactogenic hormones such as prolactin or hydrocortisone might modulate the extent and direction of cholesterol transport in MEC. This will help to determine if the apoA-I/ABCA1 complex acts predominantly as cholesterol transport mechanism relevant for the milk composition or rather as pathway in redirecting cholesterol back into bloodstream.

## Conclusions and Perspectives

The present study demonstrates the suitability of ex vivo collected and frozen MG tissues in defining the binding kinetics of ^125^I-apoA1 and ^3^H-cholesterol, and the applicability of those *ex vivo* criteria to optimize the frequently used cholesterol efflux cell culture model in terms of time and efficiency. Furthermore, the results confirmed the relevance of the apoA-I/ABCA1 complex in cholesterol transport in the MG and showed differences in apoA-I mediated efflux between the apical and basolateral sides of MeBo cells at steady state conditions. Additional studies are needed to explore a potential modulation of vectorial cholesterol transport by pregnancy-related and lactogenic hormones, and to identify the underlying intracellular signaling processes associated with apoA-I/ABCA1 activities in MEC. Together, this will help to better understand the functional impact of the apoA-I/ABCA1 pathway in cholesterol transport associated with milk formation during lactation.
